# Case Report: Identification of a novel *PRR12* variant in a Chinese boy with developmental delay and short stature

**DOI:** 10.3389/fped.2024.1367131

**Published:** 2024-05-09

**Authors:** Zhengxia Liu, Shuxia Ding, Guangwei Xu, Chunyan Fang

**Affiliations:** ^1^ Department of Neurology, Women and Children’s Hospital Affiliated to Ningbo University, Ningbo, Zhejiang, China; ^2^ Department of Endocrinology, Women and Children’s Hospital Affiliated to Ningbo University, Ningbo, Zhejiang, China; ^3^ Department of Pediatric Orthopedic, Women and Children’s Hospital Affiliated to Ningbo University, Ningbo, Zhejiang, China

**Keywords:** *PRR12*, whole exome sequencing, neurodevelopmental disorder, short stature, intellectual disability

## Abstract

Proline Rich 12 (PRR12) protein is primarily expressed in the brain and localized in the nucleus. The variants in the *PRR12* gene were reported to be related to neuroocular syndrome. Patients with *PRR12* gene presented with intellectual disability (ID), neuropsychiatric disorders, some congenital anomalies, and with or without eye abnormalities. Here, we report an 11-year-old boy with a novel *PRR12* variant c.1549_1568del, p.(Pro517Alafs*35). He was the first PRR12 deficiency patient in China and presented with ID, short stature, and mild scoliosis. He could not concentrate on his studies and was diagnosed with attention deficit hyperactivity disorder (ADHD). The insulin-like growth factor 1 (IGH-1) was low in our patient, which may be the cause of his short stature. Patients with neuroocular syndrome are rare, and further exploration is needed to understand the reason for neurodevelopmental abnormalities caused by *PRR12* variants. Our study further expands on the *PRR12* variants and presents a new case involving *PPR12* variants.

## Introduction

The Homo sapiens *PRR12* gene contains 14 exons and encodes a protein with a length of 2,036 amino acids. In addition to the canonical transcript, *PRR12* also generates a shorter 1,215 amino acids isoform through alternative splicing, which lacks exons 1–3 and most of exon 4. However, the disruption of nuclear function in the larger isoform is the underlying cause of this developmental disorder (1, 2). It is highly expressed in the human brain (3) and also contains well-conserved AT-hook binding regions, which allow proteins to bind DNA (4). Furthermore, *PRR12* is believed to be related to the formation of large protein complexes and the remodeling of chromatin (4).

The *PRR12* variant was first reported disease-causing in 2015 (4) with a female patient with a *de novo* balanced t(10;19)(q22.3;q13.33) translocation which disturbed both *ZMIZ1* and *PRR12* gene. Subsequently, the pathogenicity of *PRR12* variants was identified in patients with intellectual disability, neuropsychiatric abnormalities, and iris abnormalities in progressively larger cohort studies (1, 2, 5).

Here, we report a novel variant in *PRR12* gene in an 11-year-old boy. He was affected with short stature, intellectual disability, and developmental delay. A *de novo* variant in *PRR12* gene [NM_020719.3, c.1549_1568del, p.(Pro517Alafs*35)] was identified through whole exome sequencing. This was the first reported patient in China, and our findings further expanded the genetic spectrum for *PRR12* variants.

## Materials and methods

### Patients and consents

We collected the clinical information including the results of brain magnetic resonance imaging (MRI), developmental status assessment, vision, hearing, and other general tests. We also included the clinical phenotype in reported patients with *PRR12* gene variants in our analysis.

## Whole exome sequencing (WES)

Peripheral blood was collected from the patient for WES detection. Blood cell DNA was extracted through the genome extraction kit (Thermo Fisher) according to the manufacturer's instructions. The genome library was conducted and the targeted sequence was captured through the NovoSeq 6000 platform and sequenced using the IDT xGen Exome Research Panel capture library. The reads were aligned to the human reference genome (GRCh38/hg38) through Burrows-Wheeler Aligner (6), Samtools (7), and Picard software (version 2.14.1, https://broadinstitute.github.io/picard). The dbSNP (http://varianttools.sourceforge.net/Annotation/DbSNP), ExAC (http://exac.broadinstitute.org), and 1,000 Genomes (http://www.internationalgenome.org) databases were used to screen the potential mutations. Polyphen-2 (8), Mutation Taster (9), and SIFT (10) were used to predict the harmful variants we detected. The filtered variants were further assessed according to the American Guidelines for Medical Genetics and Genomics (ACMG) (11), and verified by Sanger sequencing. The primers were designed for the Ensemble database; the upstream and downstream primer was 5′-gggtggtggtggaggtTACC-3′ and 5′- GTGACTGGAGCGGACGAATGA-3′, respectively. The amplified products were sequenced using an ABI 3500 Genetic Analyser (Applied Biosystems).

## Results

### Clinical description

Our patient is an 11-year-old boy with global developmental delay and dysmorphic features. He was born at 35 weeks and 5 days of gestation by cesarean delivery as the first child to the healthy, non-consanguineous parents. The cesarean delivery was necessitated by liver dysfunction caused by cholestasis in the mother, and she had no history of miscarriage. The birth weight was 2.1 kg (<10th percentile), and the length was 46 cm (normal). He had no other abnormalities except low birth weight. He was able to sit alone at 9 months of age, crawl at 11 months of age, and walk at 15 months of age. His height and weight have always been below the average. Learning difficulties were discovered when he started elementary school at the age of 6, and his score on the Wechsler Intelligence Test was 70. He has been diagnosed with ADHD. He has a younger sister who is healthy and has no developmental delay. He was subsequently admitted to the hospital for short stature and developmental delay.

**Figure 1:**
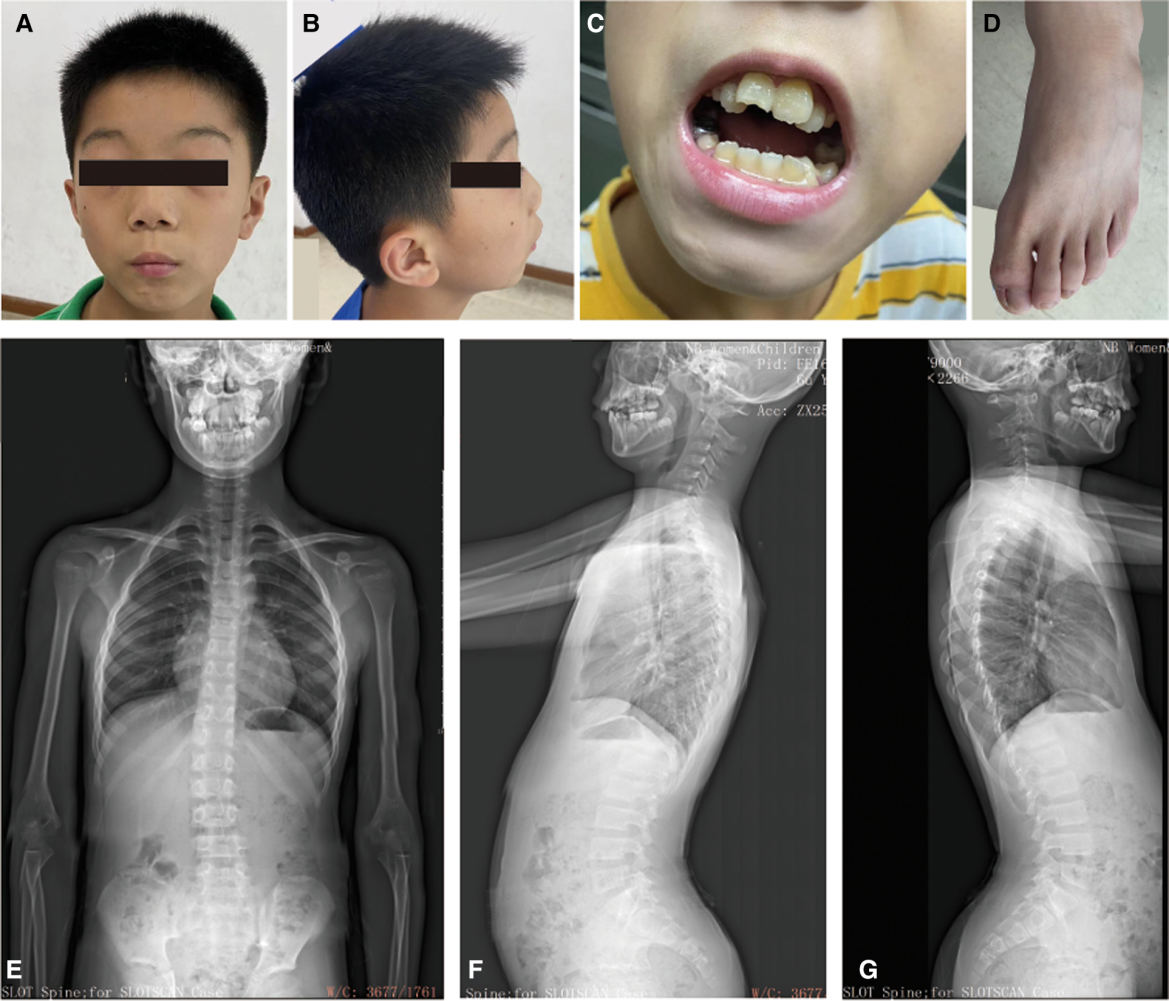
The clinical information for the patient. (**A**–**C**) Facial dysmorphology, dental malocclusion, and abnormalities in foot skin. (**D**) Bone age radiograph obtained at the age of 11 years (left hand). The Bone age corresponds to actual age. (**E**–**G**) Scoliosis x-rays showing a T12-L1 right curvature, Cobb angle of 11.5°. He has an anterior pelvic tilt, forward-leaning spine, increased thoracic kyphosis, and anterior cervical tilt.

His reactions and normal communication are normal, and he gets along well with his classmates. However, he has special features, including a low hairline, an triangular face, a wide gap between the eyebrows and the upper eyelid, small palpebral fissures, bilateral bulging eyeballs, noticeable freckles around the eyes, a low nasal bridge, interpupillary distance, a short nose with a visibly upturned tip, a small mandible, dental malocclusion, no cleft palate, bilateral low ears and excessive dermal wrinkling on the feet (Figures 1A–C). The persistent left superior vena cava was found in his heart color ultrasound examination. He has normal hearing and vision, further he was evaluated by an ophthalmologist and the evaluation was normal. Now, our patient is 11 years and 8 months old, with a bone age of 11. The height of his father and mother are 173 cm and 163 cm, respectively. The patient is short stature with a current height of 134.2 cm (−2.17SD).

The results of brain magnetic resonance imaging and B-ultrasound examination for urinary system organs were normal. Once again, the Wechsler intelligence test score was 57 in July 2023. The bone age corresponds to his actual age when he was 11 years old (Figure 1D). However, he has a mild degree of spinal scoliosis (Figures 1E–G). X-rays show a T12-L1 right curvature with a Cobb angle of 11.5°. He presents with an anterior pelvic tilt, a forward-leaning spine, an increased thoracic kyphosis, and an anterior cervical tilt. His secondary sexual characteristics have not developed, characterized by small testicles and penis (left testicle: 22 × 10 × 14 mm, right testicle: 20 × 10 × 14 mm (2023.07.16, penis: 2 cm, no pubic hair growth). His growth hormone level was normal and the IGF-1 was significantly lower (131 ng/ml) (12). Currently, the child has been treated with recombinant human growth hormone since 2023-7-29.

The patient received human growth hormone (HGH) treatment with an initial dose of 4I U/day for 4 weeks (Supplementary Table S2). Initially, there was a positive response with IGF-1 levels rising and height increasing by 0.25 cm/week. However, growth slowed after 9 weeks, despite increasing the HGH dose. Over 6 months, height increased by 3 cm and weight by 3.6 kg, but discontinuation of HGH showed no further height increase after an 8-week follow-up.

## Genetic finding

**Figure 2:**
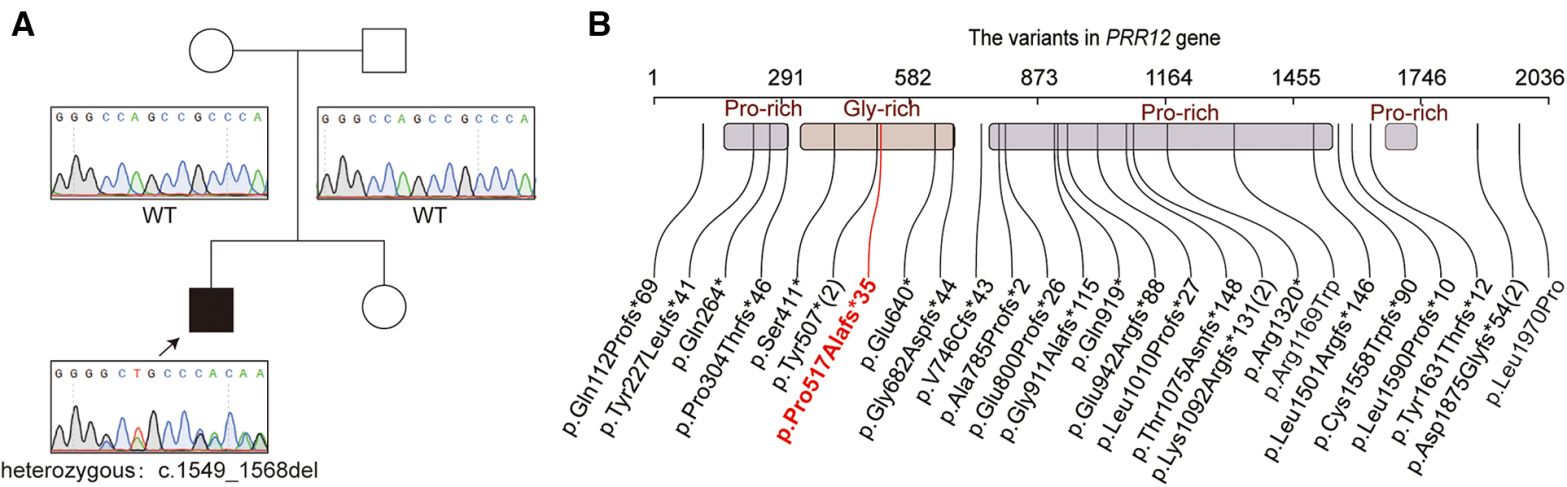
The genetic funding for the patient. (**A**) The pedigree of the family. The sanger sequencing for the patient and his parents. (**B**) Variations of the *PRR12* gene in previously reports. The variable in our patient was highlighted with red text. Variants reoccurring in patients are noted with numbers in parentheses.

Proband WES was performed to further identify the pathogenesis of our patient. He had not had any other genetic testing before this. A novel variant in the *PRR12* gene [NM_020719.3: c.1549_1568del, p.(Pro517Alafs*35)] was revealed in our patient (Figure 2A). Sanger sequencing was used to confirmed the variant in proband and parents. The result showed that the variant in our patient was *de novo* (Figure 2A). This variant is rare and is not present in public databases such as 1,000 Genomes Project, gnomAD(4.0), and ExAC. It was unique and was also not included in the ClinVar dataset. It was predicted that the protein coding would terminate prematurely. This variant was assessed to be “pathogenic” (PVS1 + PM6_Supporting + PM2_Supporting) and responsible for the clinical phenotypes of our patient according to ACMG criteria. The spectrum of *PRR12* gene variants is summarized in Figure 2B, and we review the clinical features of previous patients in Supplementary Table S1.

## Discussion

The function of the *PRR12* gene has not yet been fully characterized. The PRR12 protein contains 2,036 amino acids is highly expressed in the cerebellum, lobes, and cerebral cortex, and is mainly located in the cell nucleus (4). Furthermore, PRR12 contains special domains and epigenetic modification sites [(13), UniprotKB database]. It has two AT-hook DNA binding domains (347–359 and 381–393), which may be involved in regulating DNA-dependent processes. Genes with an AT-hook DNA binding domain, such as MECP2, are involved in human neurodevelopmental disorders (14–16). Furthermore, PRR12 has an identified epigenetic modification site, lysine acetylation site (N6-acetyllysine), suggest its potential involvement in epigenetic regulation (13). All of these indicated that PRR12 has the potential to function as a nuclear cofactor, participating in transcriptional regulation by forming regulatory protein complexes.

The variant in *PRR12* gene was first reported in a patient with neurodevelopmental disorders in 2015 (4). They found ZMIZ1/PRR12 reciprocal fusion transcripts in a girl with ID and neuropsychiatric alterations. Leduc et al. (2) further added that iris abnormalities are associated with the loss of function of *PRR12*. With further research, the range of eye abnormalities associated with *PRR12* variants has expanded. To date, 31 cases (include our case) and 28 variant types (including 1 balanced translocation and 1 microdeletion) have been reported (Figure 2C, Supplementary Table S1). A total of 24 nonsense, 2 missense variants, one balanced translocation and one microdeletion were found in *PRR12* gene.

In addition to eye abnormalities, patients with these variants also exhibit short stature, congenital anomalies affecting multiple systems, and skeletal abnormalities (1, 5). The eye abnormality was not present in our patient. Other Four patients with *PRR12* variants (1) also showed normal vision, indicating the heterogeneity of eye abnormalities in patients with *PRR12* variants. The main phenotypes in our patient were developmental delay, short stature, ID, and ADHD. He had relatively normal social skills, but learning difficulties developed when he was in elementary school. Neurological characteristics are widespread in patients with *PRR12* variants such as ASD, ADHD, and anxiety (11/31), and this is also present in our patient. This may be due to the high expression of *PRR12* in the CNS system and this suggests that *PRR12* plays an important role in early CNS development (4).

Congenital abnormalities, including heart, kidney, skeletal, and skin abnormalities were also observed in patients with *PRR12* variants. Our patient also showed congenital heart disorder with left superior vena cava. This may indicate the crucial role of PRR12 in development. The developmental delay and short stature were widespread in the reported cases (5). The IGF-1 level in the serum of our patient was 131 ng/ml, which was significantly lower compared to that of normal children of the same age (392 ng/ml). The IGF-1 is essential for childhood growth, and the growth hormone (GH)—IGF-1 axis is thought to be the central system responsible for short stature (17). IGF-1 deficiency may be associated with mutations in the IGF-I receptor (18), malnutrition, liver disease (19), and other primary diseases (20). The reason for the IGF-1 deficiency in our patient is still unknown. IGF-1 is a polypeptide produced by the liver in response to GH. GH mediates mitosis promotion by increasing IGF-1 synthesis. Therefore, we consider that in children with Neuroocular syndrome, there may be problems with GH by increasing IGF-1 synthesis, which can result in short stature and intellectual disability. Further investigation of IGF-1 in patients with *PRR12* variants is needed to determine the cause of short stature.

Our patient started HGH treatment at 4 IU/day for 4 weeks. During the subsequent treatment, the growth rate of height slowed down, and the dose of growth hormone was gradually adjusted several times, but the effect was not good. Patients who fail to achieve expected height gains while receiving growth hormone therapy require further evaluation and consideration of alternative treatment options.

In our study, we identified an 11-year-old boy with developmental delay, ID, and ADHD. Genetic testing identified a variant in the *PRR12* gene (c.1549_1568del, p.Pro517Alafs). IGF-1 level in our patient was lower than in normal children of the same age. Our study identified the first *PRR12* variant in China, which further expanded the *PRR12* cohorts and broadened the spectrum of genetic variations associated with *PRR12*.

## Data Availability

The original contributions presented in the study are publicly available. This data can be found here: ClinVar, accession number VCV002687488.1, https://www.ncbi.nlm.nih.gov/clinvar/variation/2687488/.

## Supplementary material

Supplementary Figure S1 Patient diagnosis and treatment timeline.
