# Supplementary figures and images for: Case Report: Identification of a novel PRR12 variant in a Chinese boy with developmental delay and short stature
Source: Front Pediatr. 2024 May 9;12:1367131. doi: 10.3389/fped.2024.1367131 (PMC11119739; doi:10.3389/fped.2024.1367131)

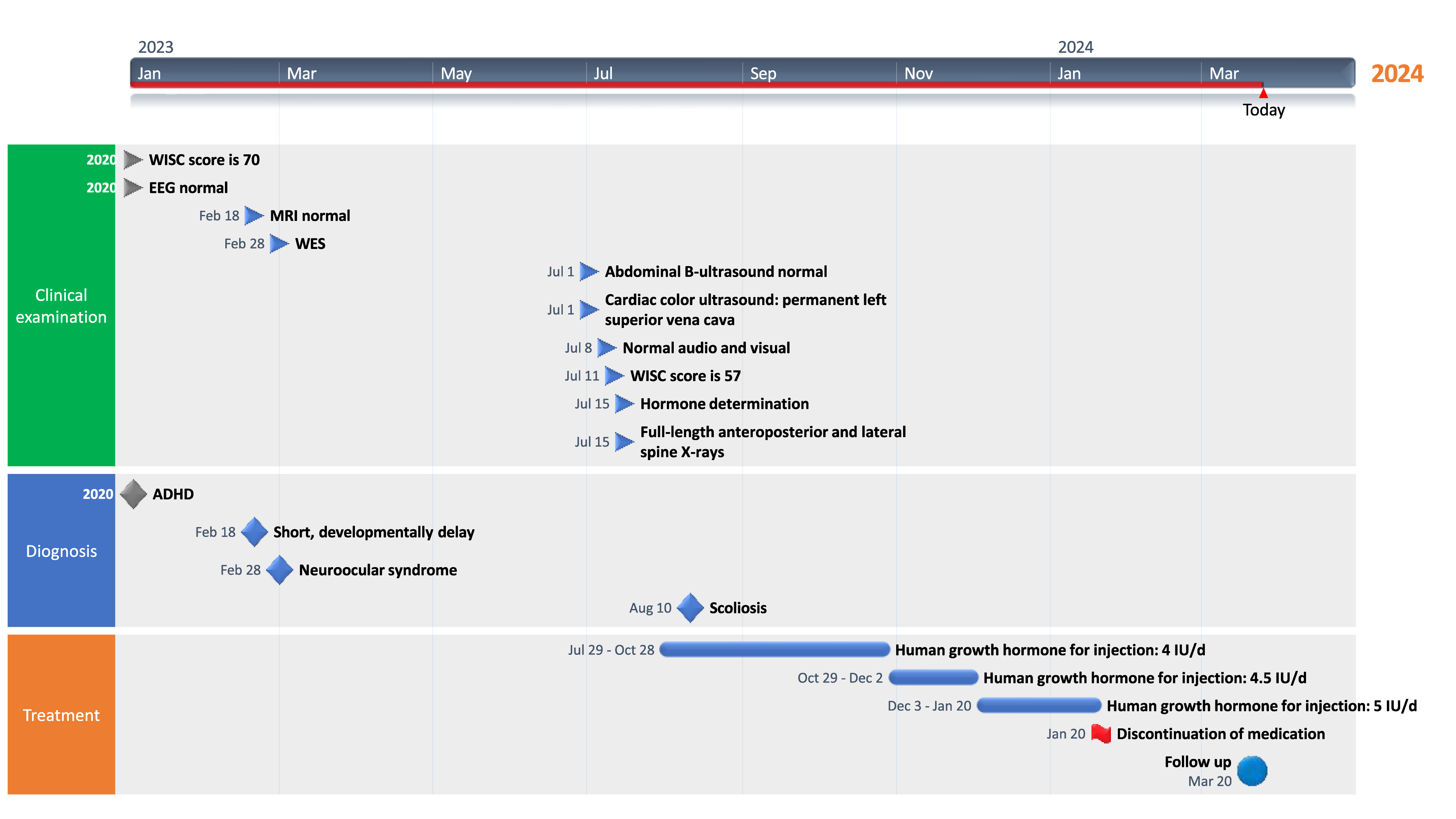

Supplement: Supplementary Figure S1 — Patient diagnosis and treatment timeline. [file Image1.tif]
